# Inconsistent prediction capability of ImmuneCells.Sig across different RNA-seq datasets

**DOI:** 10.1038/s41467-021-24303-5

**Published:** 2021-07-07

**Authors:** Xu Xiao, Canqiang Xu, Wenxian Yang, Rongshan Yu

**Affiliations:** 1 School of Informatics, Xiamen University, Xiamen, China; 2 National Institute for Data Science in Health and Medicine, Xiamen University, Xiamen, China; 3 Aginome Scientific, Xiamen, China

**Keywords:** Immunotherapy, Prognostic markers

**Arising from** D. Xiong et al. *Nature Communications* 10.1038/s41467-020-18546-x (2020)

In Xiong et al.^1^, the ImmuneCells.Sig was identified as a gene expression signature to predict response to immune checkpoint therapy (ICT) from two immune cell subpopulations that are highly enriched in tumors not responding to ICT. The derived signature achieved high prognostic value in the discovery dataset and three validation datasets, comparing to 12 previously reported ICT response signatures in melanoma patients. We found that the performance reported in the original paper can only be achieved by using predictive models trained individually on the same validation dataset. As the validation stage only reports training error, the results could be overoptimistic and the performance could drop if a model is trained on one dataset and applied on another dataset.

Xiong et al.^1^ analyzed scRNA-seq datasets from multiple studies to determine if certain types of immune cells and their subclusters are associated with ICT outcomes. They found that two immune cell subpopulations, namely, TREM2^*hi*^ macrophages and *γδ* T cells are highly enriched in the ICT non-responding tumors. Based on this finding, they further identified a gene expression signature named ImmuneCells.Sig from the scRNA-seq datasets and a bulk gene expression dataset GSE78220^2^ for the purpose of predicting response to immunotherapy. The authors verified the correlations of the signature with ICT outcomes and found that the signature achieved high prognostic value in the discovery dataset (GSE78220 AUC 0.98). ImmuneCells.Sig was then validated on three independent gene expression datasets of pretreatment melanoma (GSE91061^3^, PRJEB23709^4^, and MGSP^5^), and achieved AUC values of 0.96, 0.86, and 0.88, respectively. PRJEB23709 is further split into two sub-datasets according to the treatment scheme, and ImmuneCells.Sig achieved AUC values of 0.88 and 0.93 for the two subsets respectively. Comparisons with AUC values of 12 previously reported ICT response signatures show that ImmuneCells.Sig predicts the ICT outcomes of melanoma patients more accurately across the above four gene expression datasets of melanoma.

Our concerns arise from an important issue in the validation methodology of the prognostic values of ImmuneCells.Sig, which would prevent the generalization of its prediction capability on other datasets and hence limit its utilization value in medical practice. The validation datasets (GSE91061, PRJEB23709, and MGSP) were not used in developing the gene list of ImmuneCells.Sig. However, in the source codes that we retrieved from the Github repository released by the authors (https://github.com/donghaixiong/Immune_cells_analysis, version 2.7.4), the AUC values reported in the original paper can only be achieved by using predictive models trained individually on the same validation dataset. In such a validation setup, the reported AUC value in fact only reflects the training error rather than the test error. Hence, the result can be overoptimistic and may not truly reflect the classification accuracy if any of the trained models is applied on other external datasets.

To illustrate the effect of overfitting due to training and testing on the same dataset, we compared ImmuneCells.Sig with randomly selected gene sets following the same training procedure as used in the original paper (Methods). The average AUC values from 50 bootstrapping in all datasets are higher than 0.75 from random gene sets (Fig. 1). Notably, the highest AUC reaches 1 for GSE78220, 0.917 for GSE91061, 0.902 for PRJEB23709, and 0.835 for MGSP. Moreover, none of the random gene sets has an AUC performance lower than 0.7, indicating that training error could be misleading in reporting the prediction performance of a machine learning algorithm.

**Fig. 1:**
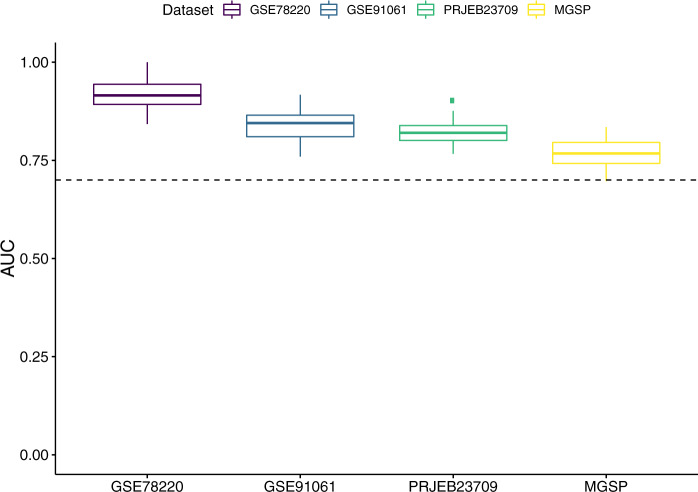
The predictive ability of a random gene set by using the same implementation scheme with Xiong et al.^1^ on four melanoma datasets. Fifty random sampling of around 100 genes among total genes in each dataset were tested faithfully using the implementation provided by the original paper. The actual number of genes used is 103 for GSE78220 and GSE91061, 101 for PRJEB23709, and 98 for MGSP. Box plots show the median (center line), upper quartiles and lower quartiles with whiskers extend to 1.5x interquartile range. The dotted line is drawn at AUC value equal to 0.7.

When applying machine learning algorithms to a real-life scenario, the generalizability of the model has to be evaluated using test data which shall not appear in the model training procedure. To this end, the datasets from which the model learns its parameters shall typically be split into three sets, namely, training, validation, and test sets. The training set is used to fit the models, the validation set is used to estimate prediction error for model selection, while the test set is used for assessment of the generalization error of the chosen model. Ideally, the test set should be kept in a “vault”, and be brought out only at the end of the data analysis^6^. Clearly, with only the performance on the training set, it is insufficient to conclude that the features can apply to other datasets due to possibilities of model overfit.

We next tested the generalization capability of ImmuneCells.Sig using the four melanoma datasets from the original paper (Methods). We found that the predictive model using features from ImmuneCell.Sig did not show robust prediction results (Fig. 2). In most cases, the testing AUC values are around 0.5, which clearly casts doubt on using the developed model in clinical applications to predict the outcome in melanoma patients receiving immunotherapy.

**Fig. 2:**
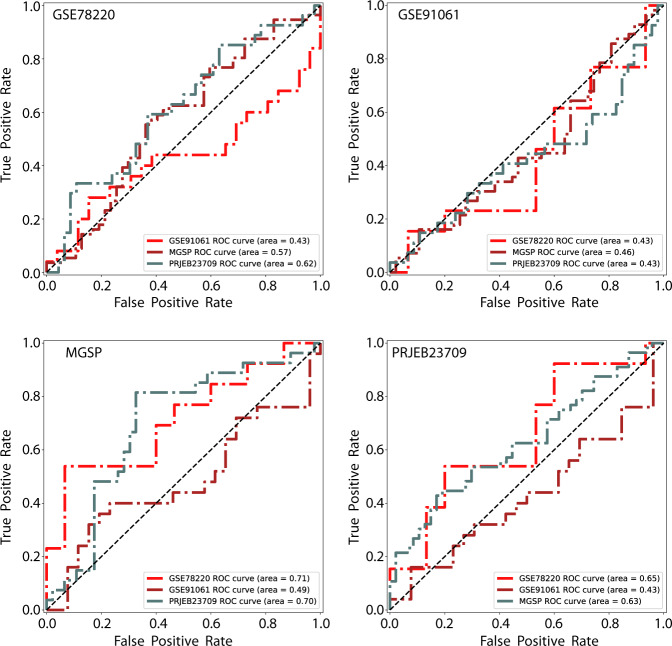
The performance of the ImmuneCells.Sig signature on predicting ICT outcomes in four melanoma patient datasets. For each ROC curve plot, the plot title refers to the training dataset for the predictive model, while the trained model was tested on the three remaining datasets.

In summary, our results do not dispute the potential immune suppressive roles of the cell subtypes identified in the original paper, or the potential predictive values of the genes in the ImmuneCells.Sig gene set. Rather, we have demonstrated that the predictive model developed in the original paper is not able to generalize across different RNA-seq datasets. Although ImmuneCells.Sig was not developed in the validation datasets, the reported predictive error on each validation dataset in the original paper is actually the training error from classification models trained on the same dataset. Therefore, the generalizability of the model was not evaluated in the original paper. We further demonstrated that ImmuneCells.Sig with the nearest-centroid classification model as described in the original paper was unable to predict ICT outcomes well on independent test sets not seen in the training set. Therefore, we conclude that there is insufficient support that ImmuneCells.Sig could be applied for clinical decision making in melanoma patients receiving immunotherapy.

## Methods

### RNA-seq expression datasets

We downloaded the preprocessed datasets GSE78220^2^, GSE91061^3^, PRJEB23709^4^, and MGSP^5^ directly from Xiong et al.^1^ (https://github.com/donghaixiong/Immune_cells_analysis). The preprocess procedure in the original paper included three steps. First, the raw RNA-seq reads were aligned to the hg19 human reference genome using Bowtie-TopHat (version 2.0.4)^7^. Then, the gene expression read counts were obtained with the htseq-count Python script from HTSeq v0.11.1 (https://htseq.readthedocs.io/en/release_0.11.1/). The read counts data were further transformed by regularized log (DESeq2 v1.28.1^8^).

### Testing with random gene sets shows the effect of overfitting

The ImmuneCells.Sig signature was discovered from the scRNA-seq datasets^9^ and a bulk gene expression dataset GSE78220^2^. A nearest-centroid classifier^10,11^ was then trained by cancerclass v1.32.0 R package^10^ to predict ICT response using the identified signature. The performance of the prediction was then evaluated by the receiver operating characteristic curves (ROC) and the area under the ROC curve (AUC).

To illustrate the effect of overfitting due to training and testing on the same dataset, we evaluated random gene sets with the same implementation. We randomly drew a set of genes from all the genes present in each gene expression dataset without considering their biological functions as a signature, and trained nearest-centroid classifiers individually on each dataset using the model fitting code provided by the original paper^1^. For fair comparison, the number of genes selected equals the number of genes of the ImmuneCells.Sig signature for each dataset, respectively (103 genes for GSE78220 and GSE91061, 101 genes for PRJEB23709, and 98 genes for MGSP). After 50 bootstrapping in all datasets, the highest AUC value could reach 1 for GSE78220, 0.917 for GSE91061, 0.902 for PRJEB23709, and 0.835 for MGSP (Fig. 1).

### The generalization capability of ImmuneCells.Sig

To test the generalization capability of the ImmuneCells.Sig signature, we changed the prediction process such that one dataset was used for training a model with the original model fitting code without modification, while the remaining independent datasets were used for testing. The predictive models using ImmuneCell.Sig did not show robust prediction results (Fig. 2). In some cases, the testing AUC values were below 0.5.

## Data Availability

All data used in this study are freely available at https://github.com/xmuyulab/ImmuneCellsSig_Comment/tree/main/data (from the original paper^1^). Four public datasets can be retrieved from Gene Expression Omnibus (GEO) under accession numbers GSE78220, GSE91061, from ENA project under accession number PRJEB23709, and MGSP from dbGaP under accession number phs000452.v3.p1.

## Code availability

All data analysis code used in this study is freely available at https://github.com/xmuyulab/ImmuneCellsSig_Comment/.
